# Diversity and flexibility of algal symbiont community in globally distributed larger benthic foraminifera of the genus *Amphistegina*

**DOI:** 10.1186/s12866-021-02299-8

**Published:** 2021-09-06

**Authors:** Martina Prazeres, T. Edward Roberts, Shadrina Fildzah Ramadhani, Steve S. Doo, Christiane Schmidt, Marleen Stuhr, Willem Renema

**Affiliations:** 1 Naturalis Biodiversity Center, Leiden, Netherlands; 2 Leibniz Centre for Tropical Marine Research (ZMT), Bremen, Germany; 3 Department of Biology, California State University, Northridge, USA; 4 MARUM, University of Bremen, Bremen, Germany; 5 University of the Ryukyus, Nishihara, Okinawa, Japan; 6 Interuniversity Institute for Marine Sciences (IUI), Eilat, Israel; 7 Bar-Ilan University (BIU), Ramat Gan, Israel

**Keywords:** Microbiome, Symbiosis, Endosymbionts, Photosymbiosis, Phylogeography, Coral reefs

## Abstract

**Background:**

Understanding the specificity and flexibility of the algal symbiosis-host association is fundamental for predicting how species occupy a diverse range of habitats. Here we assessed the algal symbiosis diversity of three species of larger benthic foraminifera from the genus *Amphistegina* and investigated the role of habitat and species identity in shaping the associated algal community.

**Results:**

We used next-generation sequencing to identify the associated algal community, and DNA barcoding to identify the diatom endosymbionts associated with species of *A. lobifera*, *A. lessonii*, and *A. radiata*, collected from shallow habitats (< 15 m) in 16 sites, ranging from the Mediterranean Sea to French Polynesia. Next-generation sequencing results showed the consistent presence of Ochrophyta as the main algal phylum associated with all species and sites analysed. A significant proportion of phylotypes were classified as Chlorophyta and Myzozoa. We uncovered unprecedented diversity of algal phylotypes found in low abundance, especially of the class Bacillariophyta (i.e., diatoms). We found a significant influence of sites rather than host identity in shaping algal communities in all species. DNA barcoding revealed the consistent presence of phylotypes classified within the order Fragilariales as the diatoms associated with *A. lobifera* and *A. lessonii*, while *A. radiata* specimens host predominately diatoms of the order Triceratiales.

**Conclusions:**

We show that local habitat is the main factor influencing the overall composition of the algal symbiont community. However, host identity and the phylogenetic relationship among hosts is relevant in shaping the specific endosymbiont diatom community, suggesting that the relationship between diatom endosymbiont and hosts plays a crucial role in the evolutionary history of the genus *Amphistegina*. The capacity of *Amphistegina* species to associate with a diverse array of diatoms, and possibly other algal groups, likely underpins the ecological success of these crucial calcifying organisms across their extensive geographic range.

**Supplementary Information:**

The online version contains supplementary material available at 10.1186/s12866-021-02299-8.

## Background

Algal symbiosis drives the normal functioning of coral reef ecosystems [1]. It plays an important role in facilitating the adaptation or acclimatisation of organisms to environmental change, and their capacity to successfully expand into new habitats [2]. The algal endosymbionts allow hosts to exploit light as an energy source in oligotrophic conditions [3], and since symbiont communities can often confer distinct physiological capacities they influence the host’s geographic distribution range or habitat preferences (e.g., [4]). For example, the symbiosis between reef-building corals and dinoflagellates is crucial for the persistence of coral reefs [5] but makes them inherently vulnerable to environmental change when this symbiosis is lost (i.e., thermally induced bleaching [6];). However, the capacity of hosts to utilise a diverse pool of symbionts may alleviate this vulnerability and provide hosts with the capacity to acclimate to ongoing ocean warming (e.g., [7, 8]). Symbiosis is influenced by complex interactions between the host, the symbionts, and the local environment [9], which shapes the fitness of the holobiont (i.e., host-symbiont complex and associated microbiome) [10].

Larger benthic foraminifera (LBF) are amongst the most common and abundant marine organisms to host algal symbionts [11] and are known to have an obligatory symbiosis with multiple groups of algae, such as rhodophytes, chlorophytes, diatoms, and dinoflagellates [12]. The ecological advantage of maintaining algal symbiosis is evident in the recurrent emergence of symbiosis in foraminifera over the past 350 My, despite repeated extinction events of symbiotic species [13]. Symbiosis has driven morphological differentiation and speciation of symbiont-bearing species along depth gradients (e.g., [14, 15]), as well as ‘horizontally’ across trophic gradients (e.g., [16]). For example, the depth distribution of diatom-bearing species is viewed as indicative of their adaptive potential to wide light intensity and spectrum [17, 18], and can utilise a wider band of the available light spectrum (reviewed in [19]).

Symbiont diversity is also closely linked to host identity and phylogeny. It has been shown that in diatom-bearing LBF of the genus *Amphistegina*, the similarities and differences in lineages of endosymbionts of four closely related species are consistent with what is known of their evolutionary histories [20]. Similarly, dinoflagellate symbionts found in the Caribbean and the Indo-Pacific show phylogenetic divergence, which is consistent with the phylogenetic relationship within their LBF hosts [21]. Some species of *Amphistegina* show a stable and persistent algal symbiosis unaffected by water quality gradients [22], while the diversity of algal symbionts in diatom-bearing nummulitids changes over depth [23]. The diversity of symbionts might also play a key role in thermal stress tolerance [24, 25], and potentially facilitates geographic range expansion in response to ocean warming [26, 27]. The presence of a consortium of diverse algal species that can be functionally relevant within different environmental conditions may include thermo-tolerant genotypes or species [22, 25]. Similar patterns of changes in algal symbiont consortiums can also be found in some species of dinoflagellate-bearing LBF, where mixed infections are common [9, 21]. Yet, it remains unclear whether symbiont community is driven by host identity (i.e., phylogenetic lineage), habitat (e.g., physicochemical conditions), or a combination of these factors, and to what extent the diversity of associated symbionts allows LBF to respond to changes in environmental conditions and expand their distribution range across shallow habitats worldwide.

Here, we utilise next-generation sequencing to examine the diversity of algal symbiont communities associated with morphotypes of *Amphistegina lobifera*, *A. lessonii* and *A. radiata* living in shallow habitats (< 15 m) across the Mediterranean, Red Sea, Indian and Pacific Oceans. *Amphistegina lobifera* and *A. radiata* rarely overlap in distribution along the depth gradient. *Amphistegina lobifera* frequently occupy shallow areas (0–12 m), and *A. radiata* is known to be a deep specialist, with abundances peaking between 30 and 90 m, and rarely occupying shallow depths [15, 28]. In contrast, *A. lessonii* regularly co-occurs with *A. lobifera* and *A. radiata*, and this overlap is largely contingent on local geography and environmental conditions of sites [15]. Additionally, *A. radiata* occurs almost exclusive on rubble, whereas *A. lobifera* and *A. lessonii* also occur epiphytically on macroalgae [18] and seagrass (e.g., [29]). Specifically, we investigate the influence of the local habitat on the composition of the algal symbiont community within individual species, but also in sites where *A. lobifera* and *A. lessonii* co-occur. We used DNA barcoding to explicitly address the level of endosymbiont specificity between different species hosting diatoms, and evaluate whether these host-endosymbiont associations are species-specific. We distinguish between “endosymbionts”, such as diatoms where a known long-term mutual relationship exists in *Amphistegina* sp. [30], and a rather flexible “algal symbiont community” with other algae taxa where the nature and duration is not yet known.

## Results

### Diversity and identity of algal symbionts

**Fig. 1:**
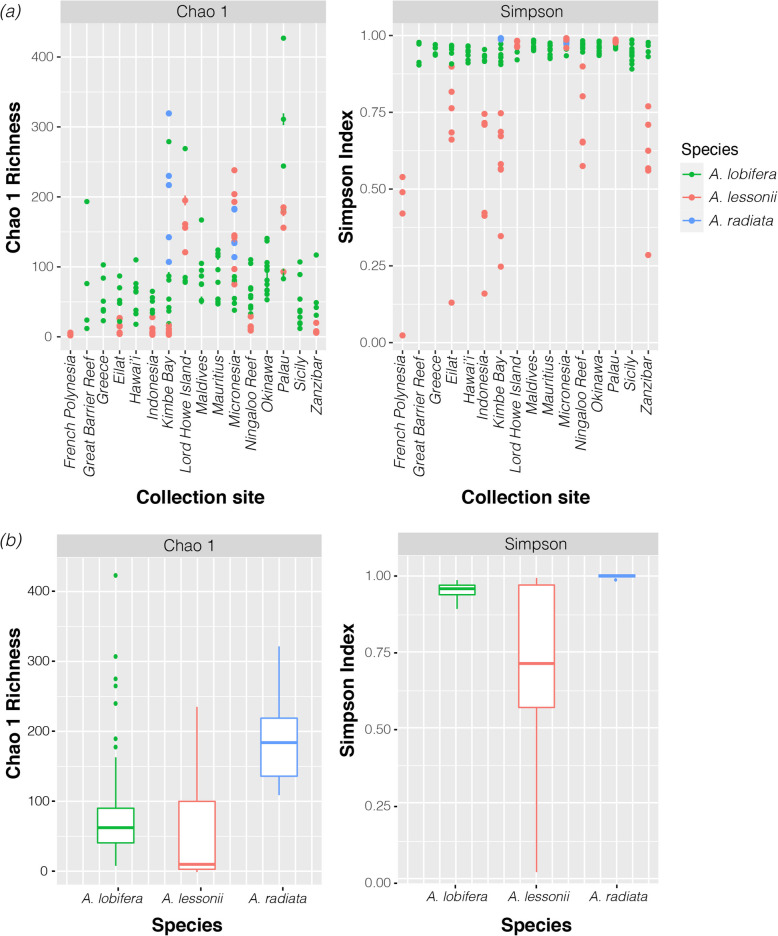
Estimated richness (Chao 1 and Simpson) of ASVs. (**a**) Estimate richness per specimens in each collection site. (**b**) Average richness per species, including all specimens collected from different sites

The algal symbiotic community of *Amphistegina* consisted of 6232 identified ASVs. After the removal of singleton and low count ASVs (< 5% summed across all samples), a total of 527 ASVs remained. Estimated total alpha diversity per specimen varied between species and among sites (Fig. 1; Table 1). Among sites, alpha diversity is consistently variable between specimens in all three species, and there is no clear pattern between diversity and site (Fig. 1a; Supplementary Table S1). The highest average diversity of ASVs was found in *A. radiata* collected from Kimbe Bay, Papua New Guinea, whereas the lowest diversity was consistently found in *A. lessonii* specimens (Fig. 1a). In contrast, Simpson’s diversity index, which incorporates evenness, is strongly constrained among sites in *A. lobifera* and *A. radiata*, but it varies between sites in *A. lessonii* (Fig. 1a). Within species, alpha diversity in *A. lessonii* is consistently lower than in *A. lobifera* and *A. radiata* (Fig. 1b). Additionally, Simpson’s index is significantly lower in *A. lessonii* than *A. lobifera* and *A. radiata* (Fig. 1b), suggesting a higher prevalence of dominant ASVs in *A. lessonii*.

**Table 1:** Kruskal-Wallis rank sum test results of diversity indices among sites and species

Term	Sites			Species		
	χ^2^	df	*p*-value	χ^2^	df	*p*-value
Chao 1	77.814	15	**< 0.01**	33.47	2	**< 0.01**
Simpson	68.761	15	**< 0.01**	39.968	2	**< 0.01**

**Fig. 2:**
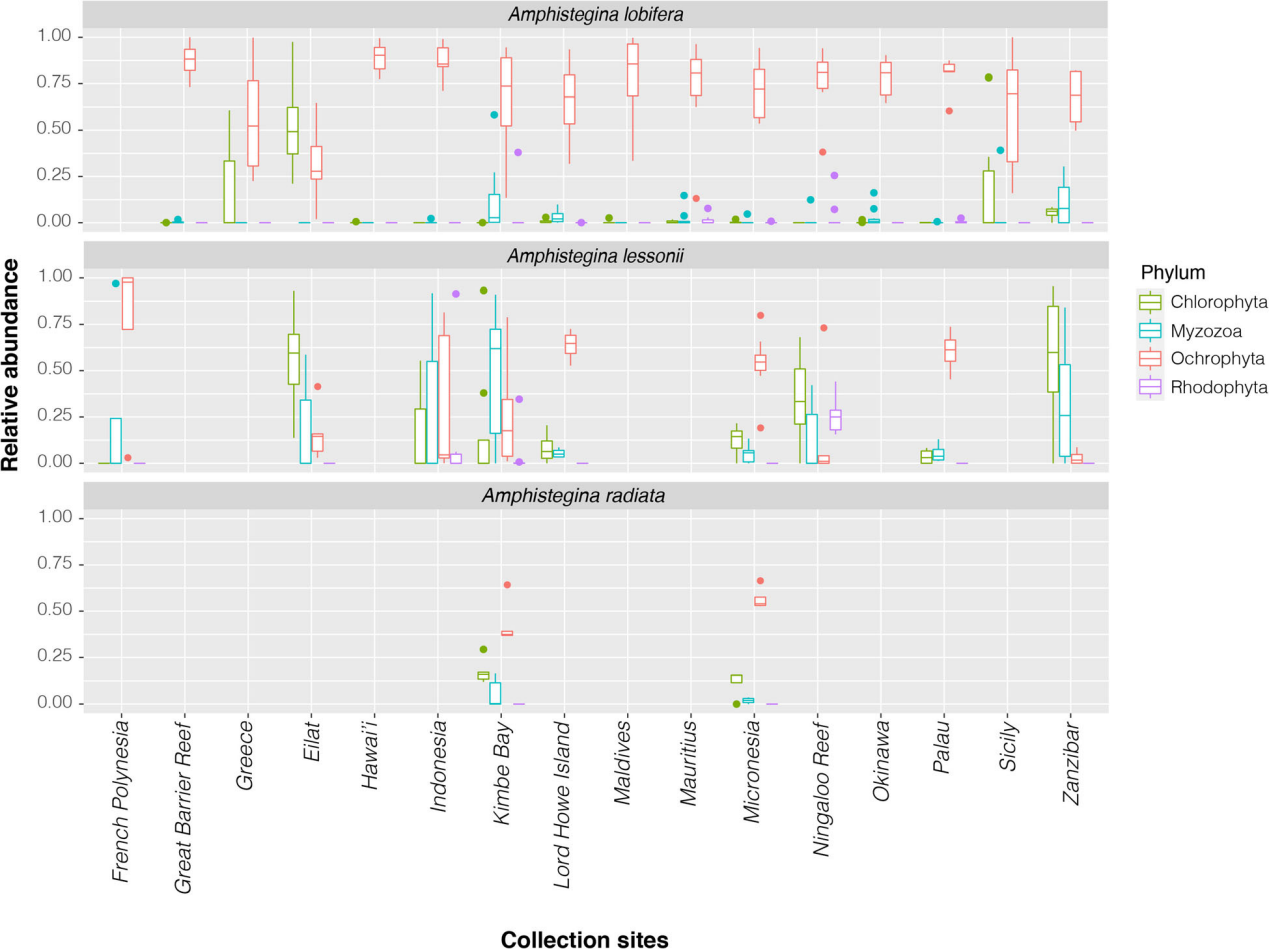
Relative abundance of algal taxa across different species and sites. (**a**) Relative abundance of algal groups classified as ‘Ochrophyta’, ‘Chlorophyta’, ‘Myzozoa’, and ‘Rhodophyta’

**Fig. 3:**
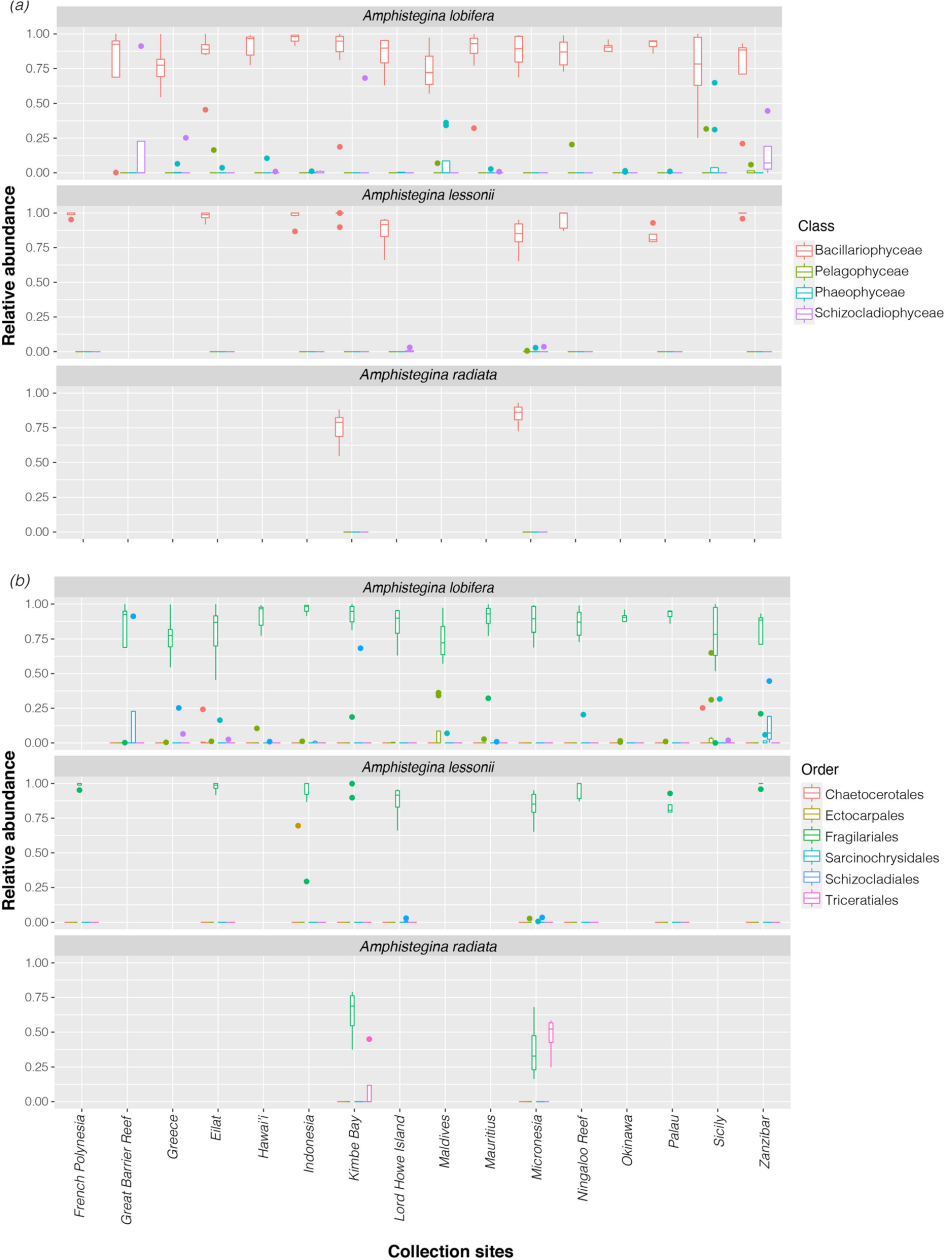
Relative abundance of (**a**) classes of ‘Ochrophyta’ and (**b**) orders of Bacillariophyceae across different species and sites

ASVs classified as Ochrophyta, which includes diatoms known to be endosymbionts of *Amphistegina*, were the most prevalent (Fig. 2). The overall relative abundance of Ochrophyta was 69.6 ± 2.4% (mean ± SEM). However, other algal phyla, such as Chlorophyta (14.8 ± 1.8%), Myzozoa (11.2 ± 1.5%), and Rhodophyta (4.3 ± 0.9%) represented a substantial proportion of the associated algal symbiont community in *Amphistegina* as well (Fig. 2; Supplementary Table S2). For example, the average relative abundance of Chlorophyta was as high as 57.3 ± 15.6% in samples of *A. lessonii* from Zanzibar, the highest abundance of an algal taxon other than diatoms (Figs. 2, 3). Within the Chlorophyta taxa, *Ostreobium* was the most abundant genus observed, particularly in *A. lessonii*. Relative abundance of *Ostreobium* reached as much as 98% of ASVs in *A. lessonii* specimens collected from Zanzibar (Supplementary Table S2). *Ostreobium* was also found in lower abundance (< 5%) in *A. lobifera* and *A. radiata* from Micronesia. Another major algal group represented in the algal community were the Myzozoa, in particular from the class Dinophyceae such as those belonging to the families Amphidiniales and Peridiniales. For example, in *A. lessonii* from Kimbe Bay, ASVs classified as Myzozoa represented a substantial proportion (51.3%) of the algal community (Supplementary Fig. S1). Lastly, ASVs classified as Rhodophyta were also present, but only in significant proportions in *A. lessonii*. While most ASVs were classified as belonging to the family Corallinales, other families such as Ceramiales were also identified (Supplementary Table S2).

In total, 3603 ASVs were classified as Ochrophyta. Within the phylum Ochrophyta, ASVs classified as class Bacillariophyceae (i.e., diatoms) were consistently present across sites and species (Fig. 3a). The most common Bacillariophyceae belonged to the order Fragilariales, which represented over 60% of identified diatoms. Common genera include *Serratifera* and *Nanofrustulum* (Supplementary Table S2). Diatoms from the order Triceratiales (genus *Orizaformis*) were found to be common within specimens of *A. radiata*, especially those specimens collected from Micronesia (24.1 ± 9.6% of the identified diatoms), which also showed the lowest relative abundance of Fragilariales (59.5 ± 3.7%). Triceratiales were rare or absent in *A. lessonii* and *A. lobifera* (Fig. 3b).

### Multivariate analysis of diversity of algal biome within and between species and sites

In all species analysed, the factor ‘Site’ captured a substantial proportion of the overall variability (Table 2). However, in all three species variability was high and inconsistent between sites. For example, variability within samples of *A. lobifera* collected from Zanzibar, Indonesia, and the Gulf of Aqaba, Eilat was low, while samples from Okinawa, Ningaloo Reef, and the Great Barrier Reef showed high variability (Supplementary Fig. S2). As a result, the factor ‘Site’ explained ~ 45% of the variability in the data. Similar to *A. lobifera*, *A. lessonii* and *A. radiata* also showed this pattern of variability, with some sites featuring more variation than others in the algal community between sites (Supplementary Figs. S3 and S4). Nevertheless, the factor ‘Site’ captured ~ 50% of the variability found in these species (Table 2).

**Table 2:** One- and Two-way Permutation ANOVA results for Bray Curtis distance matrix of algal symbiont community associated with specimens of *A. lobifera*, *A. lessonii* and *A. radiata* collected across a wide distribution range, analysed together and individually. Results are based on 1000 permutations

Term	df	SS	R-squared	Pseudo-F	*p*-value
*A. lobifera*					
Site	14	24.33	0.57	8.72	**< 0.01**
Residuals	90	17.94	0.43		
Total	104	42.28	1.00		
*A. lessonii*					
Site	8	8.56	0.36	3.10	**< 0.01**
Residuals	43	14.81	0.63		
Total	51	23.37	1.00		
*A. radiata*					
Site	1	0.48	0.46	8.77	**0.027**
Residuals	7	0.55	0.54		
Total	8	1.04	1.00		
*A. lessonii* x *A. lobifera*					
Species	1	2.47	0.11	15.91	**< 0.01**
Site	7	5.43	0.24	4.99	**< 0.01**
Species*Site	7	2.03	0.09	1.86	**< 0.01**
Residual	80	12.42	0.56		
Total	95	22.35	1.00		

**Fig. 4:**
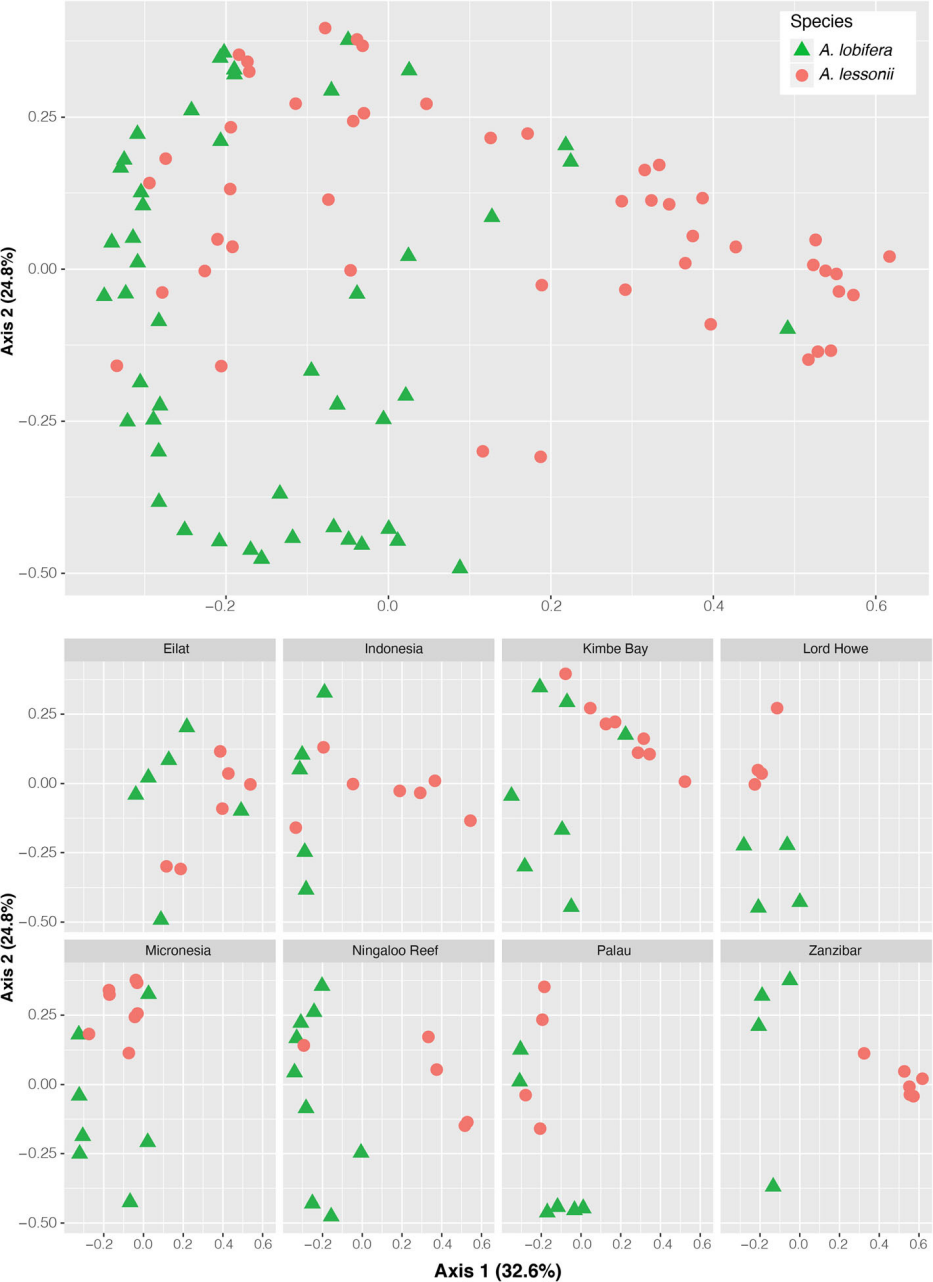
Two-dimensional plots of Principal Coordinates Analysis utilising Bray Curtis distance matrix showing differences in algal symbiont community in *A. lobifera* and *A. lessonii* collected from different sites

Comparison of algal symbiont community between *A. lobifera* and *A. lessonii* showed that despite algal symbionts being more distinct between sites than between species, variability within sites was highly uneven (Fig. 4; Table 2). As a result, the majority of variability found in our dataset could not be explained by these two factors independently nor by their interaction (~ 56%). Sites contained algal communities with low variability shared by both species (i.e., Palau), low variability with discrete species-specific communities (i.e., Zanzibar and Lod Howe Island), and high variability regardless of species identity (i.e., Kimbe Bay and Ningaloo Reef).

### Species-specificity of algal endosymbionts

**Fig. 5:**
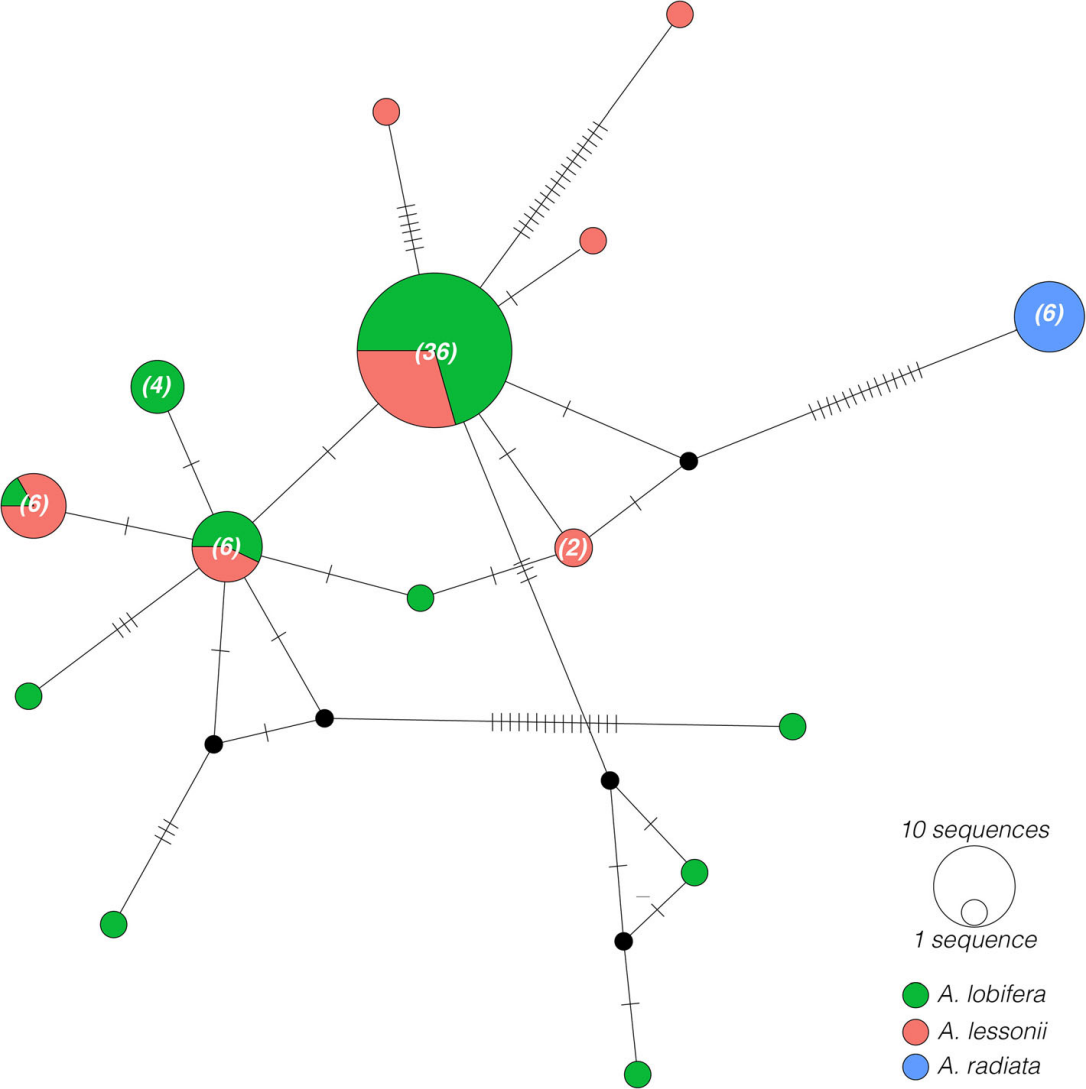
Median-joining haplotype network showing phylogenetic relationship of diatoms collected from *A. lobifera*, *A. lessonii*, and *A. radiata*. Circled areas are proportional to the number of individuals bearing a particular haplotype. If neighbouring haplotypes differ by more than a single substitution, the changes are designated as ticks. Numbers in the parentheses indicate the observed number of haplotypes

**Fig. 6:**
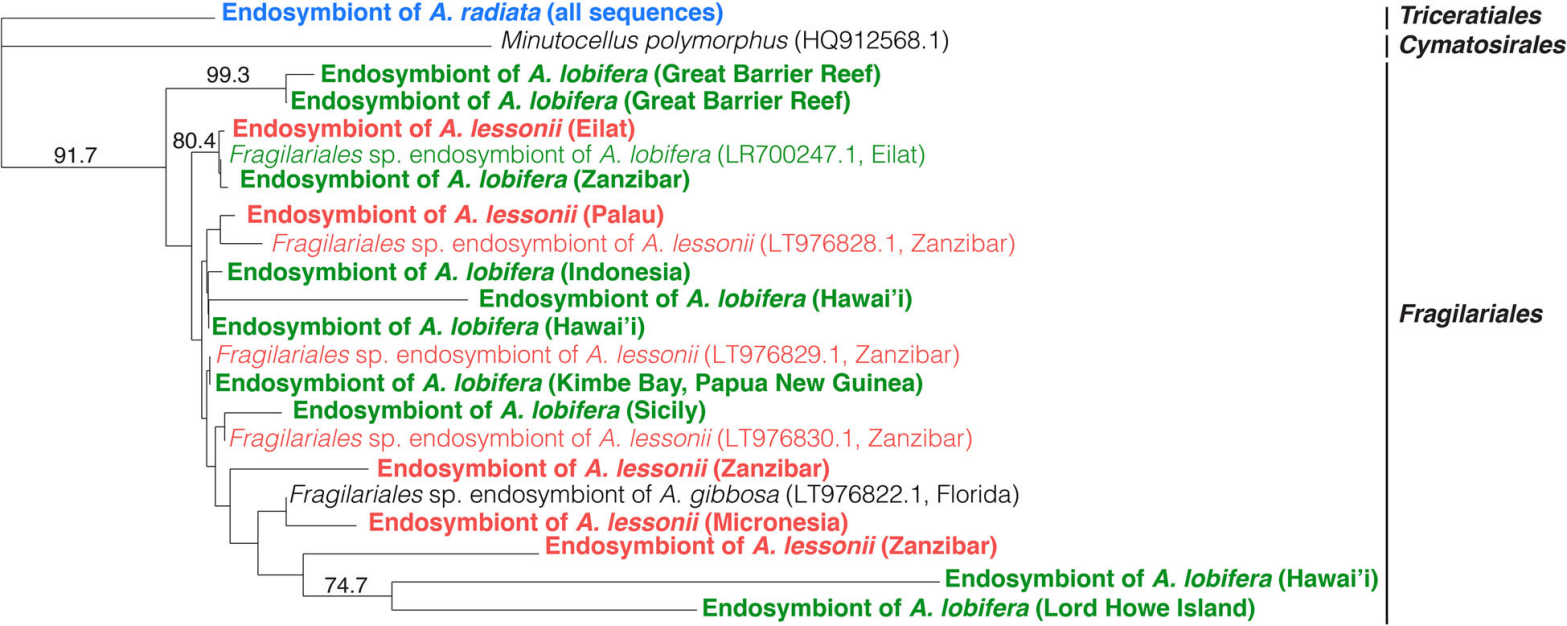
Unrooted phylogenetic tree with taxonomy assignment of unique sequences of diatom endosymbionts recovered from *A. lobifera*, *A. lessonii*, and *A. radiata*. Sequences of endosymbionts of *A. lobifera* from Eilat [31], *A. lessonii* from Zanzibar and *A. gibbosa* from the Florida Keys [24], as well as a sequence of *M. polymorphus* as an outgroup were added. Bootstrap support above 70% is given at the respective nodes

While most endosymbiont diatoms were successfully amplified and sequenced, some PCR amplicons of diatom endosymbionts of *A. lessonii* and *A. lobifera* were heterogeneous and could not be directly sequenced through Sanger sequencing. In addition, diatoms from specimens of *A. lessonii* collected from French Polynesia and *A. lobifera* collected from Greece failed to amplify using the SymSF1-1528R primer set. As a result, we ended up with 68 diatom endosymbiont sequences in our dataset (Supplementary Table S3). DNA barcoding of diatom endosymbionts revealed that *A. lobifera* and *A. lessonii* share several endosymbionts (Fig. 5), independent of their collection site, whereas specificity was observed in *A. radiata*. Both *A. lessonii* and *A. lobifera* host several haplotypes belonging to different lineages, but all haplotypes fall within the order Fragilariales. Conversely, all specimens of *A. radiata* host diatom endosymbionts primarily belonging to the order Triceratiales (Fig. 6).

## Discussion

Algal symbiont plasticity facilitates host organisms to accommodate changes in environmental conditions (e.g. [32]). Our results showed high algal symbiont flexibility in *Amphistegina*, which potentially underpins their wide distribution range and adaptation capacity (e.g., [27, 33]). The similarities and differences in symbiont associations of the three species of *Amphistegina* are primarily shaped by site at which the individual occurs rather than the identity of the species analysed in our study. This association between site and algal symbiont community is expected (e.g., [4]), but these findings reveal an unexpectedly high level of variability in the algal communities within sites, especially in *A. lobifera* and *A. lessonii*. Each site is subject to a unique set of environmental conditions, which dictate the performance of differing algal symbiont types, and consequently the fitness of the host [22, 24].

Our results show that the most common endosymbionts in *Amphistegina* belonged to Ochrophyta, more specifically diatoms of the order Fragilariales (class Bacillariophyceae), confirming and expanding on previous results [20, 22, 24, 34] and 2000 + isolations of diatoms in culture (reviewed in [35]). We unveil an unprecedented diversity of diatoms, especially in *A. lobifera* and *A. radiata*, supporting earlier morphological observations that *Amphistegina* seem to be particularly favourable to form associations with a wide range of diatom species [36]. Despite the low number of specimens collected in our study, *A. radiata* shows the highest alpha diversity among species analysed, and therefore patterns of diversity are unlikely to be an artefact of sampling effort. We also find a consistent presence of ASVs classified as Chlorophyta and Myzozoa. The nature of this relationship is unknown, and these taxa are likely to have a transient relationship with *Amphistegina*. For example, we detect a substantial presence of *Ostreobium* sp. (Chlorophyta) and ASVs belonging to Dinophyceae in *A. lessonii*, and a lesser degree in *A. lobifera* and *A. radiata*. These results add to the growing evidence that the symbiont community of LBF is far more complex than previously assumed (e.g., [22, 37]).

We demonstrate that symbiont communities are mainly dictated by their collection site. While patterns of alpha diversity are partly informed by species identity, levels of flexibility are predominantly shaped by site. This suggests a crucial role of the symbiont community as an important interface between the host and the local environment (e.g. [24, 25]) through the capacity of symbionts to assist with modulating local physicochemical conditions (e.g., [38]). Symbiont communities respond differently to varying conditions, and the high variability within sites reveals that a wide array of symbiont communities is available within most sites (Fig. 4). Conversely, the algal symbiont community is more constrained in some sites than others, raising the possibility that local availability rather than host selectivity shape the host’s symbiont community (e.g., [39]). For example, *A. lobifera* populations that occur at the edge of their geographic distribution tend to have a highly variable algal community, with high variability between specimens from the same site (e.g., Okinawa, Ningaloo Reef, Sicily), whereas in the core of their distribution a consistent algal community across specimens is more common (e.g., Palau, Indonesia, Great Barrier Reef). The ability to acquire a variety of algal symbionts possibly imbues an advantage on populations at the range expansion forefront, allowing an increased environmental tolerance provided by the symbionts (e.g., [25]). In contrast, *A. lessonii* not only showed lower alpha diversity of algal symbionts compared to both other *Amphistegina* species but also less variability among sites. As a result, we were unable to find a universal core algal biome across all *Amphistegina* species and sites analysed, and only a local-scale species-specific core biome was detected, further supporting the hypothesis that the composition of the algal symbiont community is largely shaped by site [22]. Ultimately, our results suggest that local microhabitat, and the environmental factors associated with it, are likely to impose the strongest influence on the availability of algal taxa and how hosts acquire their symbionts.

The ability to acquire a wide array of algal taxa (i.e., flexibility) or constraints in algal acquisition (i.e., specificity) also appear to vary according to the taxonomic scale being analysed. Other diatom-bearing genera of foraminifera are hosts to diatoms of families other than Fragilariales. For example, *Pararotalia calcariformata* primarily hosts *M. polymorphus* [34], which belongs to the family Cymatosirales. Whereas nummulitids such as *Heterostegina*, *Cycloclypeus*, and *Nummulites* host diatoms belonging to the family Thalassionematales [23], and the diversity of diatoms within nummulitids is often low [36]. A similar pattern is also found in dinoflagellate-bearing species. The majority of dinoflagellate-bearing genera consistently retain a specific symbiont group [40]. Conversely, analysis of algal symbiont communities along a natural environmental gradient showed that the dinoflagellate-bearing *M. vertebralis* has highly flexible symbiosis at species level [41]. Similar to our results, different populations of *Marginopora* select their algal symbionts according to their local environment (e.g., [41]). This means that specificity may be more prevalent at higher taxonomic levels (i.e., class to family), and increasingly flexible as taxonomic scale decreases (i.e., genus and species).

Another possibility to consider is that some algal taxa (i.e., less abundant diatom taxa and other algal groups found in low abundance) are used as food by the hosts or epiphytes. As food, algal species could be retained in the cytoplasm of the host and show up in the sequences despite being functionally irrelevant for the symbiont pool. It has been demonstrated that *Amphistegina* relies on photosynthesis for most of its energy requirements [42]. Yet, *Amphistegina* is known to utilise heterotrophic feeding on algae and bacteria for nutrient acquisition [43]. Species within the algal symbiont community found in low abundance (between 1 and 5%) detected in our study, and previous culturing studies (reviewed in [30]), could play an important role as associates, but they are likely to be used as food as opposed to as to be primary endosymbionts [20]. The presence of other algal groups such as Chlorophyta, Myzozoa, and Rhodophyta can be due to the common association of *Amphistegina* with different types of substrates. *Amphistegina* species can live as epiphytes on several substrates ranging from turf algae on coral rubble in the Great Barrier Reef [28, 44] to growing on macroalgae *Jania* sp. in the Mediterranean Sea [45] as well as on other types of algae where they find optimal light and nutrient conditions for growth (e.g., [46]).

Finally, the presence of different diatom groups may reflect how species of *Amphistegina* occupy different depth ranges and habitats. *Amphistegina radiata* tends to be more abundant at greater depths and commonly occurs within reef rubble [15, 28],. *Amphistegina lobifera* is constrained to shallow habitats [15, 28], but has an exceptional capacity of extending its distributional range [26] because it can retain its thermal tolerance as an invading species [45] and ability to continue to thrive under a wide range of *p*CO_2_ conditions [31]. It is also suggested that *A. radiata* belongs to a separate lineage that evolved independently from *A. lessonii* and *A. lobifera* [47]. We found that *A. radiata* specimens host phylotypes belonging to the diatom order Triceratiales (class Bacillariophyceae; Fig. 3), which was absent in *A. lobifera* and *A. lessonii*. These results further support previous barcoding analysis that also showed *A. radiata* can host diatoms other than Fragilariales [20]. It seems that the preference for Fragilariales or Triceratiales (Figs. 4, 6) is consistent with morphological adaptation to light and habitat preference between the two groups (*A. lobifera*-*A. lessonii* and *A. radiata* [47, 48];). Nevertheless, the presence of Triceratiales in *A. radiata*, in both Sanger and NGS sequencing datasets, and the absence of this algal group in *A. lobifera* and *A. lessonii* collected from the same sites (i.e., Micronesia and Kimbe Bay, Papua New Guinea) suggests a species-specificity relationship between diatom endosymbionts within *Amphistegina*. Previous studies have also shown *A. gibbosa*, which is a species assumed to belong to the same lineage as *A. radiata* and is restricted to the Atlantic Ocean, to be associated with a single sequence type of *Fragilariales* [24] or very low symbiont diversity [20]. The limited geographic distribution of *A. gibbosa* might contribute to the reduced number of symbionts found as in the Caribbean the diversity of symbionts available is likely to be lower (e.g., [49]). Overall, this reflects what is known of the evolutionary histories of *Amphistegina* [47].

## Conclusions

The analysis of our global-scale dataset shows that the three most common shallow-water species of *Amphistegina* primarily host diatoms but can also associate with a wide range of other algal groups. Our results further suggest that while the presence of an algal symbiont community is crucial to the host, the identity of species within the community is not, and that the nature of the symbiont community is primarily shaped and evolutionarily reinforced by local habitat. Nevertheless, the differences in lineages of diatom endosymbionts likely reflect the phylogenetic relationship between hosts. The differences in their diatom symbionts, which are evident at the order level, represent the possibility that the two *Amphistegina* lineages (i.e., *A. lobifera*-*A. lessonii* and *A. radiata*) independently acquired, and co-evolved with, their symbionts. The ability to acquire a diverse and flexible array of algal species within these two orders might underlie their ubiquitous presence throughout the Indo-Pacific and the Red Sea, and most recently their successful invasion of the Mediterranean Sea [50]. Hence, *Amphistegina* populations can respond to shifts in environmental conditions and occupy a wide range of habitats, making them well-suited to further adapt to a changing climate.

## Methods

### Species, study sites and collection of samples

**Fig. 7:**
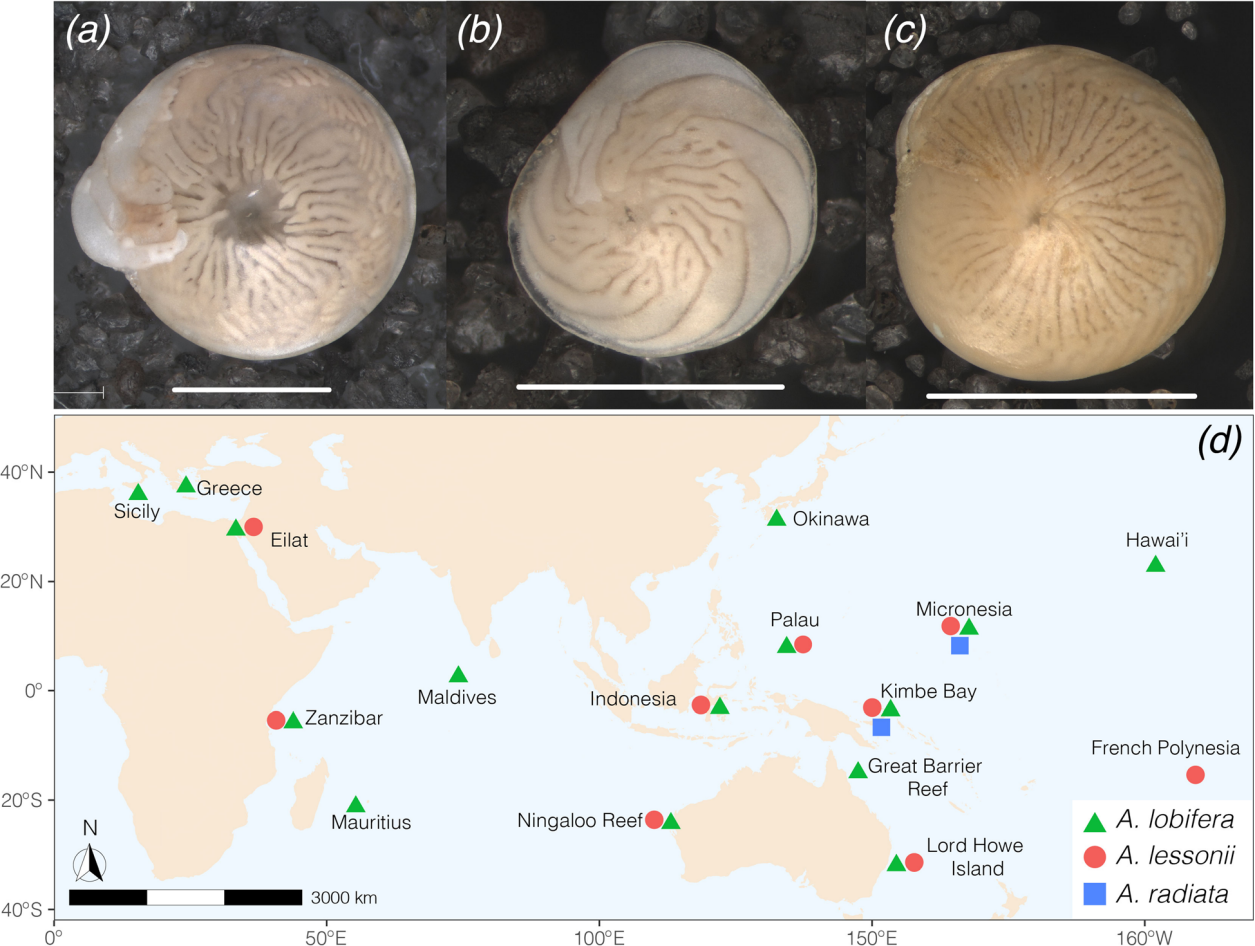
Specimens of (**a**) *Amphistegina lobifera*, (**b**) *A. lessonii*, and (**c**) *A. radiata* collected from the same habitat in Kimbe Bay, Papua New Guinea. Scale bars represent 1 mm in (**a**) and (**b**), and 2 mm in (**c**). Note that specimens were preserved in 96% ethanol, and therefore symbiont pigment colour shown here does not represent natural coloration. (**d**) Sampling sites across the Mediterranean (Sicily and Greece), Red Sea (Eilat), Indian Ocean (Maldives, Mauritius, Zanzibar, and Ningaloo Reef), and Pacific Ocean (Indonesia, Kimbe Bay in Papua New Guinea, Okinawa in Japan, Palau, Micronesia, Hawai’i, Great Barrier Reef, and Lord Howe Island). Red, green, and blue circles represent collection sites for *A. lessonii*, *A. lobifera*, and *A. radiata*, respectively. Map was generated in R using the packages *ggspatial 1.1.4* [51] and *rnaturalearthdata 0.1.0* [52]. ﻿Vector map data from http://www.naturalearthdata.com

Live adult specimens of *Amphistegina lobifera*, *A. lessonii*, and *A. radiata* were collected across a broad geographic range encompassing the Mediterranean Sea, Red Sea, Indian Ocean, and Pacific Ocean (Fig. 7; Table 3) including 16 reef sites. These sites capture a wide range of environmental conditions and span the known geographic distribution of these species [53]. However, not all species were retrieved from all sites (Fig. 7). For this study, samples were collected from shallow areas of the reef slope (< 12 m water depth) by snorkelers or SCUBA divers following previously described methods [33]. Briefly, pieces of reef rubble containing the targeted species were collected, scrubbed, and specimens picked out and immediately placed in 96% ethanol or air dried after sampling for further analysis. All specimens per site were collected from the same rubble sample.

**Table 3:** Collection sites, coordinates (lat; long), and depth of samples collection, and number of specimens per species analysed in our study

Collection site	Lat; long	Depth (m)	*A. lobifera*	*A. lessonii*	*A. radiata*
Sicily, Italy	36.74470; 15.11820	1	10		
Vravona, Greece	37.9218111; 24.0141889	1	9		
Maldives	1.92499; 73.39966	8	8		
Mauritius	−20.28666; 57.36098	2	8		
Okinawa, Japan	26.65182; 127.85624	0.1	14		
Eilat, Israel	29.5023; 34.918	2	5	7	
Zanzibar	−6.145603; 39.12445	2	4	8	
Ningaloo Reef, Australia	−23.15007; 113.75268	5	10	5	
Makassar, Indonesia	−4.71898; 119.25418	5	6	7	
Kimbe Bay, Papua New Guinea	−5.42119; 150.09434	3	8	8	5
Pohnpei, Micronesia	6.758169; 157.91721	5	8	5	4
Palau	7.30573; 134.50250	3	7	6	
Hawai’i, USA	21.64144; −157.91791	2	8		
Great Barrier Reef, Australia	−14.68383; 145.47186	8	4		
Lord Howe Island, Australia	−31.51960; 159.05620	5	9	4	
Mo’orea, French Polynesia	−17.47583; 149.82222	8		5	

### Samples processing and DNA extraction

Between four and twelve specimens per species per site were selected. In the laboratory, specimens were cleaned with 96% molecular grade ethanol under a stereomicroscope, and individual photos were taken utilising a stacking microscope (Zeiss SteREO Discovery V12). Individuals were subsequently placed in tubes containing 96% molecular grade ethanol for further wash and removal of any contamination on the shell. Individuals were air dried, then placed in individual tubes containing 200 μl of lysis buffer with Proteinase K. DNA extractions were conducted using the QIAamp® DNA Micro kit (Qiagen, Germany) according to manufacturer’s instructions. DNA concentration was measured using the DropSense96 platform (Trinean, Belgium). Total DNA concentration was standardised to 2 ng per μl across all samples.

### Library preparation, next-generation sequencing, and sequences analysis

Following quality control post DNA extraction, a subset containing 177 specimens were retained for library preparation (Table 1). To identify the algal symbiont community in *Amphistegina*, we amplified the hypervariable V4 region of the 18S SSU rRNA [54] utilising the universal 18S eukaryotic primers TAReuk454FWD (5′- CCAGCASCYGCGGTAATTCC − 3′) and TAReukREV3 (5′- ACTTTCGTTCTTGATYRA -3′) [55] with Nextera™ tags (Illumina, USA). All reactions were performed in 20 μl volumes containing 1x TaqMan™ Environmental Master Mix 2.0 (ThermoFisher, USA), 100 pmol of each primer, and approximately 6 ng of template DNA. This hypervariable region was amplified utilising the following conditions: initial denaturation at 95 °C for 10 min, followed by 40 cycles of 30 s denaturation at 95 °C, annealing at 52 °C for 45 s, and final extension at 72 °C for 1 min, and ended with a final extension at 72 °C for 5 min. Libraries were visualised by a 1% agarose gel electrophoresis stained with ethidium bromide. In total, amplification was successful for 175 specimens. Positive libraries were purified using a magnetic-beads based NucleoMag® NGS clean-up kit following the manufacturer’s instructions manual. Each purified library was then barcoded with unique Nextera™ labels in a second PCR reaction as follows: initial denaturation at 95 °C for 10 min, followed by 30 cycles of 30 s denaturation at 95 °C, annealing at 52 °C for 45 s, and final extension at 72 °C for 1 min, and ended with a final extension at 72 °C for 5 min. Size distribution of libraries was checked using the capillary electrophoresis in QIAxcel (Qiagen, Germany). Afterwards, libraries were normalised and pooled using the QIAgility system (Qiagen, Germany). Finally, the quality of the library products was assessed and standardised on a Bioanalyzer 2100 (Agilent) using a High Sensitivity DNA Chip. Libraries were sequenced utilising the Illumina MiSeq platform using the 2 × 300 bp paired-end protocol yielding paired-end reads that overlap almost completely. Sequencing was conducted by BaseClear (Leiden, Netherlands). Two negative control samples were used to monitor any contamination during DNA extraction and PCR amplifications, however no quantifiable DNA was detected for further analysis. A single negative control containing 18.2 Ω MilliQ H_2_O was used during library preparation and sequenced. The obtained .fasta files containing all amplicon sequences including the negative control sample were deposited to NCBI Sequence Read Archive accession number PRJNA602222. We only used samples with more than 5000 reads. Sequence data were processed using the statistical program R v3.6.1 [56], using the DADA2 workflow described in detail by Callahan et al. [57, 58]. Briefly, forward and reverse sequences lacking adaptors and primer sequences were checked for quality, trimmed, and filtered to remove low-quality sequence reads. Quality score cut-off point was determined based on the quality of both forward and reverse sequence reads, maintaining the recommended overlap for merging the sequences. The DADA2 method was utilised for barcoding filtering, de-replication, chimeric identification and removal, and merging pair-end reads. DADA2’s error model automatically filters out singletons, removing them before the subsequent sample inference step. Sample inference was performed using the inferred error model. Afterwards, an amplicon sequence variant (ASV) table was constructed, which is an analogue of the traditional Operational Taxonomic Unit (OTU). A total of 10,425 ASVs was retained after chimera removal. Sequences were then aligned, and ASVs defined at 95% similarity against the curated 18S SILVA v132 database [59]. Any sequences that were not assigned at the phylum level were filtered out of the dataset. Phylogenetic tree was constructed using the inferred ASV table without chimeras. A multiple-alignment was performed using the *decipher* package [60] in R. Subsequently, the phylogenetic tree was constructed by first building a neighbour-joining tree, and then using this tree as a starting point to fit a GTR + G + I (Generalised time-reversible with Gamma rate variation) maximum likelihood tree using the *phangorn* [61] package in R.

### Statistical analyses of next-generation sequencing dataset

Statistical analyses and graphical representations were performed in R v.3.6.1 [56]. Differences in the algal symbiont community associated with *A. lessonii*, *A. lobifera*, and *A. radiata* specimens collected from different reef sites were analysed using the packages *phyloseq* [62], *vegan* [63], and *microbiome* [64]. For this purpose, only ASVs classified as Ochrophyta, Chlorophyta, Rhodophyta, and Myzozoa, which are known symbionts in LBF [19], were retained in our dataset for further analyses. Our final dataset consisted of 6232 ASVs.

We calculated diversity indices such as Chao 1 [65], which we used to project estimates of taxonomic richness within each specimen (i.e., alpha diversity), and Simpson index that evaluates evenness and richness of a given specimen [66]. Indices were calculated using ASVs. We compared significant differences in diversity indices among species and sites by performing a rank sum Kruskal-Wallis test. Relative abundance was calculated, and only ASVs present in at least 5% summed across all samples were retained to minimise the influence of rare and incidental ASVs for the subsequent analyses. The relative abundance of algal phyla for each species and collection site was calculated, and the average relative abundance of ASVs was calculated and used to generate a heat-map using the package *ampvis2* [67]. We then squared-root transformed the abundance data, and analysed the effect of ‘Site’ in the diversity of algal symbiont community for each species individually through a One-way Permutational Multivariate ANOVA (PERMANOVA) using Bray-Curtis resemblance matrices. For the eight sampling sites where *A. lobifera* and *A. lessonii* co-occur, we tested differences within and between species and sites using a Two-Way PERMANOVA. For this analysis, we merged ASVs belonging to the same high taxonomic level (i.e., phylum-level) to reduce noise or excess features. ‘Site’ and ‘Species’ were employed as fixed factors. PERMANOVA outcomes were based on 1000 permutations using Type I Sums of Squares, and permutation of residuals under reduced model. PERMANOVAs were performed using the function *adonis2* in the *vegan* package. Homogeneity of multivariate dispersions was confirmed for the fixed factors ‘Site’ and ‘Species’ using the permutational test *betadisper* in the package *vegan*, to confirm that PERMANOVA results were not due to differences in group dispersions, but due to differences in algal community. An unconstrained Principal Coordination Analysis (PCoA) was used as a visual representation of the compositional differences among algal communities associated with *Amphistegina* populations from different collection sites, using the Bray Curtis distance matrix.

### DNA barcoding diatom endosymbionts

In addition to the identification of algal symbiont community utilising NGS, we identified the algal endosymbionts of *Amphistegina* via DNA barcoding utilising a subset of samples. Therefore, a fragment of ~ 480 bp of the 3′ end of the 18S SSU rRNA of algal symbionts was obtained from aliquots of the same DNA extractions using the PHUSION® Hot-start II polymerase (Thermo Fisher Scientific, USA), with the specific algal forward primer SymSF1 (5′-GGTTAATTCCGTTAACGAACGAGA-3′) coupled with the universal eukaryotic reversed primer 1528R (5′-TGATCCTTCTGCAGGTTCACCTAC-3′). Amplification was checked through the migration of the PCR product on a 1% agarose gel stained with SYBR Safe (Thermo Fisher Scientific, USA). PCR products were directly sequenced in both forward and reverse directions in the ABI 3730xl DNA Analyzer (Applied Biosystems, USA) by BaseClear (Leiden, Netherlands). All chromatograms were carefully checked for quality, primers trimmed, de novo assembled, and aligned with MAFFT v.7 [68] with default options in Geneious Prime v2020.0.3. Sequence alignments were imported into PopART [69], and median-joining networks [70] were used for analysis and visualisation of the genetic structure of diatoms between hosts. Finally, within the dataset, identical sequences were identified in ALTER [71] and collapsed into a single haplotype. Subsequently, sequences were blasted on GenBank (http://www.ncbi.nlm.nih.gov/BLAST) using default settings and top-hit matches used for identification at the order level. Phylogenetic analyses of unique sequences were performed using maximum likelihood under the generalised time reversible substitution model with gamma distribution. In addition to unique sequences identified in our dataset, additional unique sequences of endosymbionts of *A. lobifera*, *A. lessonii*, and *A. gibbosa* published previously [24, 34], as well as a sequence of *M. polymorphus* covering the same SSU fragment was added as an outgroup (NCBI accession number HQ912568.1). Calculation of bootstrap support values in the resulting unrooted tree was based on 1,000 pseudo-replicates in Geneious Prime v2020.0.3. The resulting tree was visualised with iTOL v4 [72].

## Supplementary Information

**Additional file 1.** Summary of diversity indices (Chao 1 and Simpson) of ASVs for all specimens analysed.

**Additional file 2.** Raw read counts and taxonomy of ASVs identified for all specimens.

**Additional file 3.** Sequence of diatoms identified using Sanger sequencing (primer set SymSF1-1528R) that were utilised to generate figures 5 and 6.

**Additional file 4 **: **Supplementary Fig. S1.** Heatmap of relative abundance of ASVs grouped by phylum for each species collected from different sites. Scale was log-10 transformed. **Supplementary Fig. S2.** Two-dimensional plots of Principal Coordinates Analysis utilising Bray Curtis distance matrix showing differences in algal symbiont community in *A. lobifera* collected from different sites. **Supplementary Fig. S3.** Two-dimensional plots of Principal Coordinates Analysis utilising Bray Curtis distance matrix showing differences in algal symbiont community in *A. lessonii* collected from different sites. **Supplementary Fig. S4.** Two-dimensional plots of Principal Coordinates Analysis utilising Bray Curtis distance matrix showing differences in algal symbiont community in *A. radiata* collected from different sites.

## Data Availability

Next generation sequencing data can be accessed at NCBI Sequence Read Archive (http://www.ncbi.nlm.nih.gov/sra) with accession number PRJNA602222. DNA barcoding sequencing data can be accessed through NCBI GenBank accession numbers MT792950-MT793017. Other data that support the findings of this study are available as electronic supplementary material within the manuscript.

## Abbreviations

LBF: Larger benthic foraminifera
18S SSU rRNA: 18S small subunit ribosomal RNA
PCR: Polymerase Chain Reaction
OTU: Operational Taxonomic Unit
ASV: Amplicon sequence variant
One-way PERMANOVA: One-way Permutational Analysis of Variance
Two-way PERMANOVA: Two-way Permutational Analysis of Variance
PCoA: Principal Correspondence Analysis
