# Supplementary material for: Diversity and flexibility of algal symbiont community in globally distributed larger benthic foraminifera of the genus Amphistegina
Source: BMC Microbiol. 2021 Sep 6;21:243. doi: 10.1186/s12866-021-02299-8 (PMC8422653; doi:10.1186/s12866-021-02299-8)
Supplement: Supplementary file 4 — Additional file 4 : Supplementary Fig. S1. Heatmap of relative abundance of ASVs grouped by phylum for each species collected from different sites. Scale was log-10 transformed. Supplementary Fig. S2. Two-dimensional plots of Principal Coordinates Analysis utilising Bray Curtis distance matrix showing differences in algal symbiont community in A. lobifera collected from different sites. Supplementary Fig. S3. Two-dimensional plots of Principal Coordinates Analysis utilising Bray Curtis distance matrix showing differences in algal symbiont community in A. lessonii collected from different sites. Supplementary Fig. S4. Two-dimensional plots of Principal Coordinates Analysis utilising Bray Curtis distance matrix showing differences in algal symbiont community in A. radiata collected from different sites. [file 12866_2021_2299_MOESM4_ESM.docx]

**Diversity and flexibility of algal symbiosis in globally distributed shallow-water large benthic foraminifera of the genus *Amphistegina***

Martina Prazeres^1,*^, T. Edward Roberts^1^, Shadrina Fildzah Ramadhani^1^, Steve S. Doo^2,3^, Christiane Schmidt^4,5^, Marleen Stuhr^2,6,7^, Willem Renema^1^

^1^ Naturalis Biodiversity Center, Leiden, Netherlands

^2^ Leibniz Centre for Tropical Marine Research (ZMT), Bremen, Germany

^3^ Department of Biology, California State University, Northridge, United States of America

^4^ MARUM, University of Bremen, Bremen, Germany

^5^ University of the Ryukyus, Nishihara, Okinawa, Japan

^6^ Interuniversity Institute for Marine Sciences (IUI), Eilat, Israel

^7^ Bar-Ilan University (BIU), Ramat Gan, Israel

^*^*Corresponding author*: [martina.prazeres@naturalis.nl](mailto:martina.prazeres@naturalis.nl)

**Supplementary material**

**Supplementary Figure S1.** Heatmap of relative abundance of ASVs grouped by phylum for each species collected from different sites. Scale was log-10 transformed.

**Supplementary Figure S2.** Two-dimensional plots of Principal Coordinates Analysis utilising Bray Curtis distance matrix showing differences in algal symbiont community in *A. lobifera* collected from different sites.

**Supplementary Figure S3.** Two-dimensional plots of Principal Coordinates Analysis utilising Bray Curtis distance matrix showing differences in algal symbiont community in *A. lessonii* collected from different sites.

**Supplementary Figure S4.** Two-dimensional plots of Principal Coordinates Analysis utilising Bray Curtis distance matrix showing differences in algal symbiont community in *A. radiata* collected from different sites.

**Supplementary Figure S1**


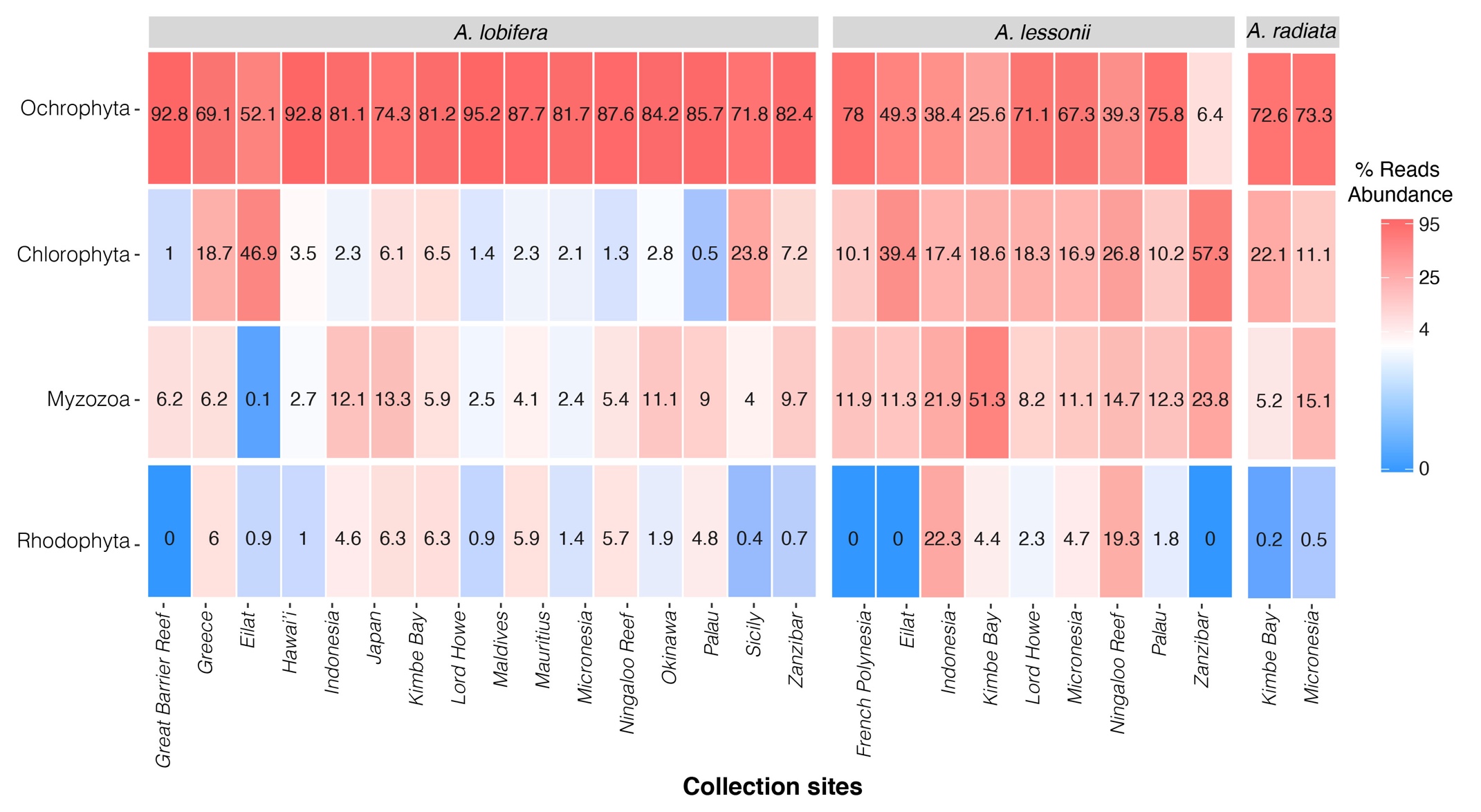


**Supplementary Figure S2**

**
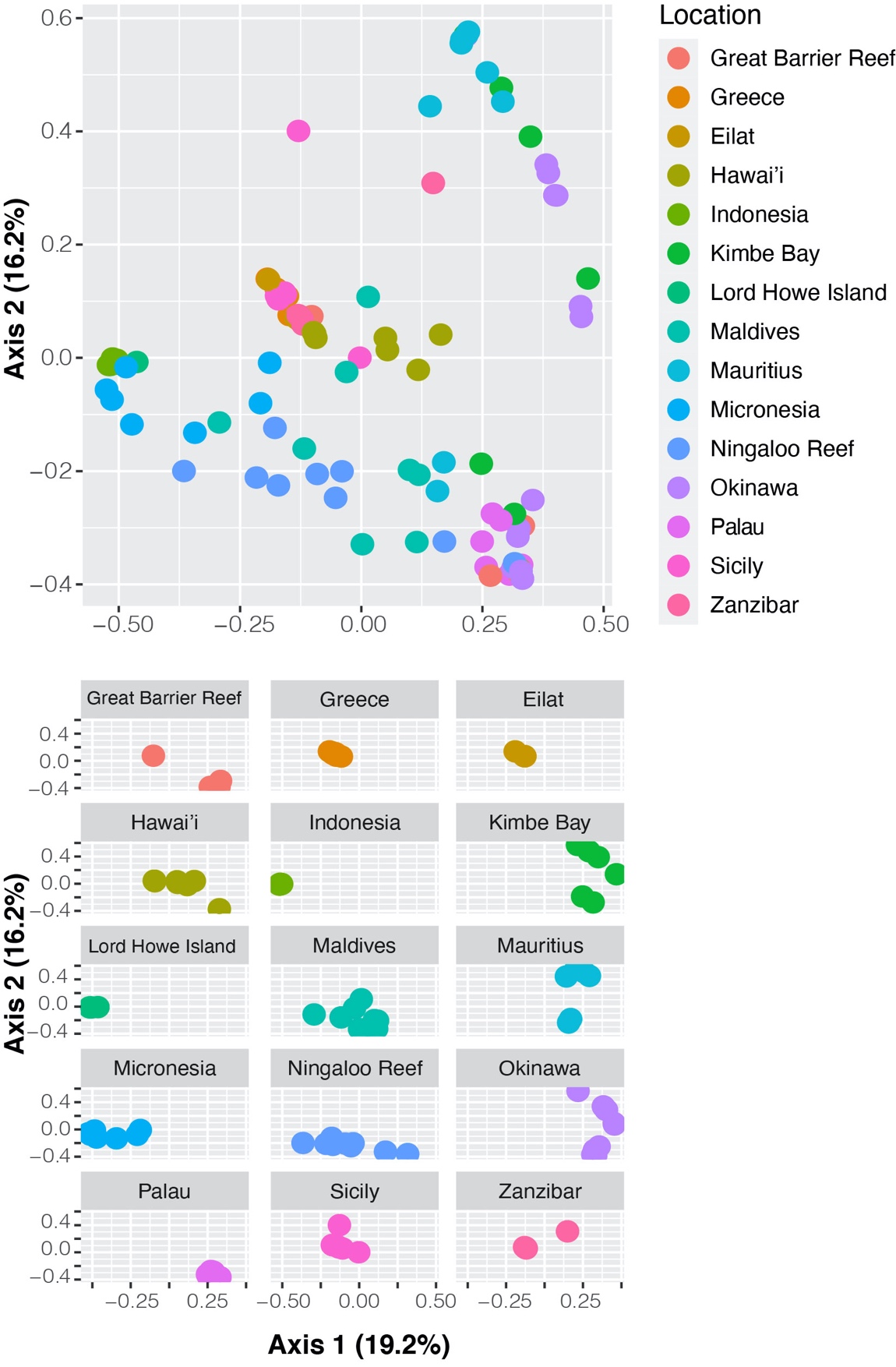
**

**Supplementary Figure S3**

**
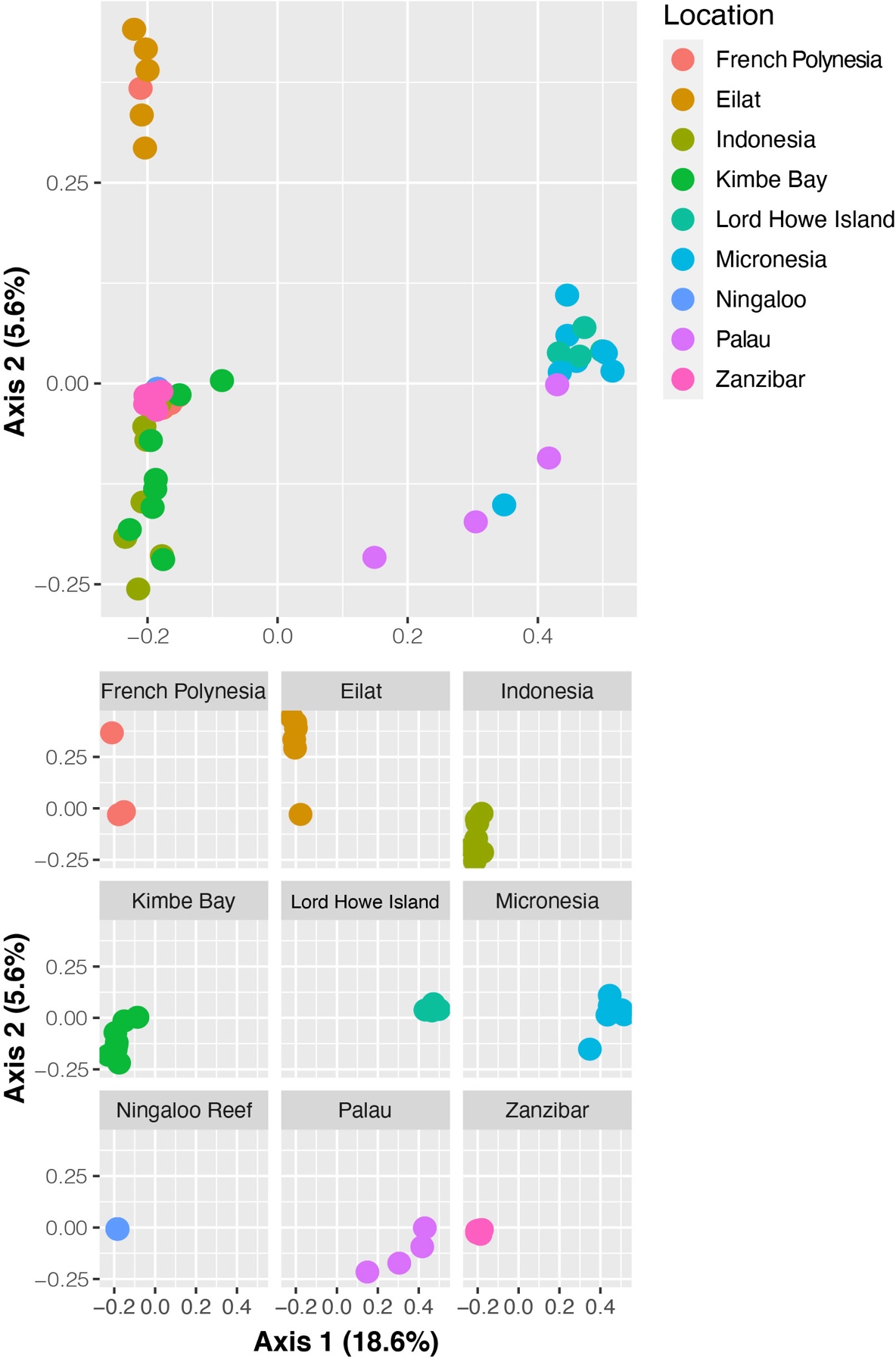
**

**Supplementary Figure S4**

**
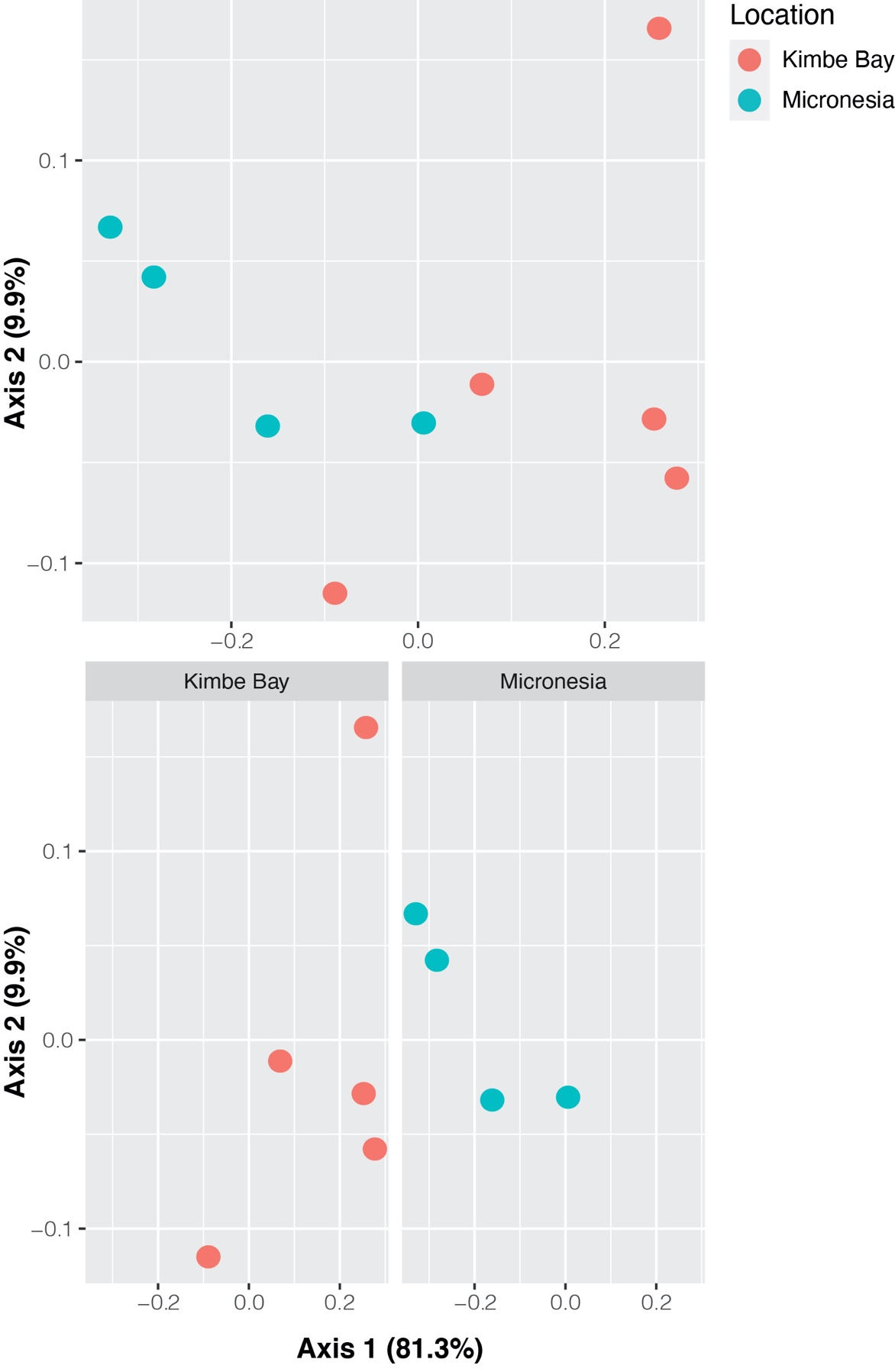
**
